# IL-1β induces IL-6 production and increases invasiveness and estrogen-independent growth in a TG2-dependent manner in human breast cancer cells

**DOI:** 10.1186/s12885-016-2746-7

**Published:** 2016-09-08

**Authors:** Keunhee Oh, Ok-Young Lee, Yeonju Park, Myung Won Seo, Dong-Sup Lee

**Affiliations:** 1 Laboratory of Immunology and Cancer Biology, Department of Biomedical Sciences, Transplantation Research Institute, Seoul National University College of Medicine, 103 Daehak-ro Jongno-gu, Seoul, Korea; 2 PharmAbcine, Inc., #461-8, DaejeonBioventure Town, Jeonmin-dong, Yusung-gu, Daejeon, Korea

**Keywords:** Luminal-type breast cancer cell, Hormone-independent, IL-1β, IL-6, Transglutaminase 2

## Abstract

**Background:**

We previously reported that IL-6 and transglutaminase 2 (TG2) were expressed in more aggressive basal-like breast cancer cells, and TG2 and IL-6 expression gave these cells stem-cell-like phenotypes, increased invasive ability, and increased metastatic potential. In the present study, the underlying mechanism by which IL-6 production is induced in luminal-type breast cancer cells was evaluated, and TG2 overexpression, IL-1β stimulation, and IL-6 expression were found to give cancerous cells a hormone-independent phenotype.

**Methods:**

Luminal-type breast cancer cells (MCF7 cells) were stably transfected with TG2. To evaluate the requirement for IL-6 neogenesis, MCF7 cells were stimulated with various cytokines. To evaluate tumorigenesis, cancer cells were grown in a three-dimensional culture system and grafted into the mammary fat pads of NOD/scid/IL-2Rγ^−/−^ mice.

**Results:**

IL-1β induced IL-6 production in TG2-expressing MCF7 cells through an NF-kB-, PI3K-, and JNK-dependent mechanism. IL-1β increased stem-cell-like phenotypes, invasiveness, survival in a three-dimensional culture model, and estrogen-independent tumor growth of TG2-expressing MCF7 cells, which was attenuated by either anti-IL-6 or anti-IL-1β antibody treatment.

**Conclusion:**

Within the inflammatory tumor microenvironment, IL-1β increases luminal-type breast cancer cell aggressiveness by stimulating IL-6 production through a TG2-dependent mechanism.

**Electronic supplementary material:**

The online version of this article (doi:10.1186/s12885-016-2746-7) contains supplementary material, which is available to authorized users.

## Background

In women, the most prevalent cancer, and the cancer associated with the most deaths worldwide, is breast cancer [1]. Despite great improvements in early disease detection and treatment, about one-third of patients will relapse with distant metastasis [2, 3]. Although the overall disease-free-survival of breast cancer patients has increased tremendously, the therapeutic options for recurrent and metastasized breast cancer are limited [4, 5]. Therefore, a detailed understanding of the molecular mechanisms underlying breast cancer aggressiveness is important to develop novel therapeutics for recurrent and metastatic breast cancers.

Due to the development of targeted therapies such as tamoxifen, and the more highly differentiated status of their cells, estrogen receptor (ER)-positive breast cancers have a lower recurrence rate during the initial 5 years after diagnosis compared to ER-negative breast cancers. However, the recurrence risk for ER-positive breast cancers increases continuously, and after 15 years the risk for both subtypes appears to be about equal [6, 7]. Thus, the recurrence of cancer cells is also an important health problem for ER-positive breast cancers. Among several explanations for tumor recurrence, cancer stem cells (CSCs) are the most fascinating and relevant. Due to their stem-cell characteristics, CSCs have tumor-initiating capabilities and are drug- and radiation-resistant [3], and thus are believed to persist as distinct populations in tumors and be associated with drug resistance, tumor recurrence, and metastasis [3, 8].

Interleukin-6 (IL-6) and downstream STAT3 signaling are implicated in inflammation-induced oncogenesis, particularly in the intestine [9]. In breast cancer, elevated serum IL-6 correlates with poor patient survival, and IL-6 expression induction in ER-positive breast cancer cells confers an epithelial-mesenchymal transition (EMT) phenotype [10]. IL-6 induces aggressive features in stem/progenitor cells from normal and malignant human mammary gland tissue [11]. Moreover, several studies have shown that IL-6/STAT3 signaling is required for the growth of CD44^+^CD24^−^ stem-cell-like breast cancer cells and for the dynamic equilibrium of breast CSCs [12, 13].

Transglutaminase 2 (TG2) is a calcium-dependent enzyme that catalyzes the cross-linking of proteins. Irreversible cross-linking of extracellular matrix (ECM) proteins by secreted TG2 is important for promoting the net accumulation of ECM molecules [14]. TG2 also activates NF-kB signaling via the polymerization of IkB and TG2 and, consequently, mediates inflammation, cancer stem cell phenotypes, and the EMT phenomenon [15, 16]. Recently, we showed that the TG2-NF-kB-IL-6 pathway in breast cancer cells is important in enhanced tumor progression and distant metastasis [17]. All basal B and some basal A cells, but not less aggressive luminal- or Her-2-type cells, were found to express both TG2 and IL-6, and expression of these genes was found to correlate with one another. A knockdown (KD) of TG2 in breast cancer cells expressing high levels of TG2 reduced IL-6 expression, moreover, TG2 KD and IL-6 KD cells did not exhibit a stem-cell-like phenotype and were unable to form tumor spheres, grow in vivo, or metastasize at distant sites in xenograft models [17].

In this study, the effects of TG2 overexpression in luminal-type TG2 non-expressing breast cancer cells on IL-6 production and aggression were examined. Simple overexpression of TG2 in MCF7 luminal-type breast cancer cells did not lead to IL-6 expression, so we then investigated additional signaling pathways that may elicit cancer cell aggressiveness through IL-6 induction. IL-1β induced IL-6 production in breast cancer cells in a TG2-dependent manner, and other cytokines and growth factors including TGF-β, TNF-α, and EGF potentiated the effect of IL-1β on IL-6 expression. In breast cancer cells, TG2 overexpression conferred EMT and stem-cell-like phenotypes, and IL-1β treatment increased stem-cell-like phenotypes, invasion, and estrogen-independent tumor growth in a TG2-dependent manner, which was attenuated by either anti-IL-6 or anti-IL-1β antibody treatment. IL-1β induced IL-6 production from TG2 overexpressing MCF7 breast cancer cells in an NF-kB-, PI3K-, and JNK-dependent manner. Thus, within the inflammatory tumor microenvironment, IL-1β, together with other cytokines and growth factor signals, increases breast cancer cell aggressiveness in a TG2-dependent manner.

## Methods

### Cell lines

Human breast carcinoma cells (MCF7 (ATCC HTB-22)) were purchased from the American Type Culture Collection (Manassas, VA) and maintained in Dulbecco’s Modified Eagle Medium (DMEM) (WelGENE, Daegu, South Korea) supplemented with 10 % heat-inactivated fetal bovine serum (FBS) (GIBCO, Grand Island, NY) and 1 % antibiotics (100 U/ml penicillin and 100 μg/ml streptomycin) at 37 °C in a humidified 5 % CO_2_ atmosphere. TG2-expressing MCF7 cells (MCF7_TG2 cells) were established by transfection with the pcDNA3.1_TG2 construct using PromoFectin (PromoKine, Heidelberg, Germany), according to the manufacturer’s instructions. Control cells were transfected with the pcDNA3.1 vector only. Stably transfected clones were established by selection with G418 (Sigma-Aldrich, St. Louis, MO) at a concentration of 500 μg/ml for 3 weeks. TG2-expressing MCF7 (MCF7_TG2 cells) clones were selected by Western blot assay.

### RNA analysis

Total RNA was isolated using an RNeasy kit (QIAGEN; 74104). cDNA was generated from 1 μg of total RNA by reverse transcription from the Moloney murine leukemia virus (M-MLV) (TAKARA, Shiga, Japan), and subjected to PCR. The following primer pairs were used for PCR: GAPDH: 5′- GGTGAAGGTCGGAGTCAACG-3′ and 5′-CAAAGTTGTCATGGATGACC-3′; Snail2: 5′-GAGCATACAGCCCCATCACT-3′ and 5′-GCAGTGAGGGCAAGAAAAAG-3′; TIMP1: 5′-AATTCCGACCTCGTCATCAG -3′ and 5′-TGCAGTTTTCCAGCAATGAG-3′; TIMP2: 5′-AAAGCGGTCAGTGAGAAGGA-3′ and 5′-CTTCTTTCCTCCAACGTCCA-3′; TIMP3: 5′-CTGACAGGTCGCGTCTATGA-3′ and 5′-GGCGTAGTGTTTGGACTGGT-3′. PCR products were analyzed by 1.5 % agarose gel electrophoresis.

### Flow cytometry

To analyze CD24 and CD44 expression in cultured MCF7 cells, cells were detached with 10 mM EDTA and stained with fluorescein isothiocyanate-conjugated anti-CD24 mAb (BD Pharmingen, San Jose, CA) and phycoerythrin-conjugated anti-CD44 mAb (BD Pharmingen). They were then analyzed using a FACSCalibur flow cytometer (BD Biosciences, San Jose, CA) and FlowJo software (Tree Star, Ashland, OR).

### ELISA

In total, 2 × 10^4^ cells were plated on a 48-well plate and allowed to adhere overnight. The medium was then replaced, and cells were permitted to grow for 24 or 48 h. Cells were treated with IL-1β (10 ng/ml) alone or in the presence of TGFβ (10 ng/ml), EGF (10 ng/ml), or TNFα (10 ng/ml) for 48 h and secreted IL-6 levels in culture supernatants were measured by ELISA. The following signaling inhibitors were added to some cultures 1 h before IL-1β treatment; IRAK1/4 inhibitor (20 μM; Calbiochem), NF-kB inhibitor (Bay-117082, 10 μM; Calbiochem), JNK inhibitor (SP600125, 10 μM; Calbiochem), ERK inhibitor (PD98059, 10 μM; Alomone labs, Jerusalem, Israel), p38 MAPK inhibitor (SB209580, 10 μM; Cell signaling), and PI3K inhibitor (LY294002, 10 μM; Alomone labs). Supernatants were collected and assayed for IL-6 by ELISA. For IL-6 detection, anti-human IL-6 (eBioscience, San Jose, CA) was used as the capture antibody, biotinylated anti-human IL-6 (eBioscience) in 0.1 % BSA in PBS/T as the detection antibody, and recombinant IL-6 (eBioscience) as the standard. All assays were performed in triplicate and were repeated two or three times under independent conditions. Data are presented as means ± SDs.

### Invasion assay

Matrigel matrix solution (250 μg/ml, Matrigel™ Basement Membrane Matrix, BD Bioscience) was applied to each Transwell (FALCON). Cells (1 × 10^5^) were seeded into the upper chamber of the Transwell and the lower chamber was filled with collagen matrix (5 μg/ml). Invasion assays were carried out for 48 h. Non-invading cells on top of the matrix were removed by rubbing with a moistened cotton swab. Invading cells on the lower surface of the Matrigel matrix were fixed with 4 % PFA and stained with 0.2 % crystal violet. Cells were counted using ImageJ software (version 1.46). In some experiments blocking anti-IL-6 antibody (10 μg/ml, eBioscience) was used.

### Three-dimensional (3D) culture tumor growth assay

Cells were cultured on Matrigel (BD Biosciences) for three-dimensional (3D) culture for 9 days. The 3D culture was setup using the on-top method as previously described [18]. A lower layer of matrix (6 mg/ml of Matrigel) was gelled for 30 min at 37 °C before adding the cell/matrix suspension. Cells were seeded in complete DMEM medium containing 1 mg/ml Matrigel. The medium was changed every second or third day, and cultures were kept at 37 °C in a humidified 5 % CO_2_ atmosphere.

### Tumor models

To assess the tumorigenicity of the cancer cells, human breast cancer MCF7_Cont and MCF7_TG2 cells (1 × 10^6^ /mouse) were injected into the mammary fat pads of 8-week-old NOD/scid/IL-2Rγ^−/−^ (NSG) mice (Jackson Laboratory, Bar Harbor, ME). Tumor growth was monitored after every other injection. To investigate the anti-breast cancer (MCF7_TG2 cells) effects, blocking anti-IL-6 antibody (100 μg/mouse; eBioscience) or blocking anti-IL-1β antibody (100 μg/mouse; eBioscience) was injected intraperitoneally every third day, starting 1 day after tumor inoculation. To inhibit TG2 activity, mice were treated with cysteamine (CyM, 40 mg/kg/day, i.p., Sigma-Aldrich) starting 1 day after tumor inoculation. Mice were bred and maintained in pathogen-free conditions at the animal facility of Seoul National University College of Medicine. All animal experiments were performed with the approval of the institutional animal care and use committee of Seoul National University (authorization no. SNU05050203).

### Western blot analysis

Cells were harvested in lysis solution containing 50 mM Tris/HCl (pH 7.6), 1 % NP40, 150 mM NaCl, 2 mM EDTA, 100 μM PMSF, a protease inhibitor cocktail (Roche Applied Science, Basel, Switzerland), and a phosphatase inhibitor (Sigma-Aldrich). After incubation on ice for 30 min, cellular debris was removed by centrifugation (10 min, 4 °C). Proteins (10 μg) were separated by SDS-PAGE and then transferred to a polyvinylidene difluoride membrane. The following antibodies were used: anti-β-actin (Sigma-Aldrich), anti-TG2 (Neomarkers, Fremont, CA), anti-E-cadherin (Santa Cruz Biotechnology, Santa Cruz, CA), N-Cadherin (Santa Cruz Biotechnology), anti-phospho-NF-kB p65 (S276) (Cell Signaling, Danvers, MA), anti- NF-kB p65 (Cell Signaling), anti-I-kB (Santa Cruz Biotechnology), anti-phospho-JNK (Santa Cruz Biotechnology), anti-JNK (Santa Cruz Biotechnology), anti-IRAK1 (Cell Signaling), anti-IRAK2 (Cell Signaling), and anti-TRAF6 (Santa Cruz Biotechnology).

### Immunofluorescence microscopy

Anti-IRAK (Cell Signaling) and anti-F-actin (Abcam, Cambridge, MA, USA) primary antibodies and Alexa 488-conjugated anti-rabbit-IgG and Alexa 546-conjugated anti-mouse-IgG secondary antibodies (all from Invitrogen, Carlsbad, CA, USA) were used. Image acquisition and processing were performed using confocal fluorescence microscopes and the FV10-ASW 2.0 Viewer (Olympus, Center Valley, PA, USA).

### NF-kB activity assay

MCF7_Cont and MCF7_TG2 cells were co-transfected with p3kB-Luc and pRL-TK reporter constructs (Promega, Madison, WI) for 24 h then treated with IL-1β (10 ng/nl) for 18 h. Luciferase activity was assayed using a kit (Promega) with a Victor3 plate reader (Perkin Elmer, Waltham, MA) and normalized against renilla luciferase activity.

### Statistical analyses

A two-tailed Student’s *t*-test was used to compare measurements for pairs of samples. Two-way analysis of variance (ANOVA) and Bonferroni *post hoc* tests were used to compare tumor volume between the two groups. All analyses were performed using SPSS software (SPSS Inc.).

## Results

### TG2 overexpression in breast cancer cells results in EMT and stem-cell-like phenotypes

**Fig. 1:**
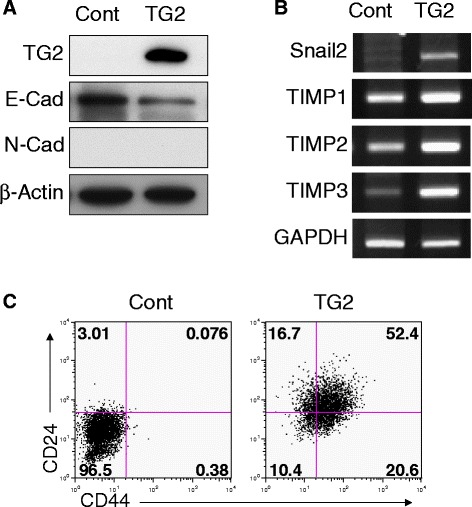
TG2 overexpression of breast cancer cells revealed EMT and stem-cell-like phenotypes. MCF7 luminal-type breast cancer cells were stably transfected with TG2 (TG2) and control vector (Cont) and EMT and stem-cell markers were compared using Western blot (**a**), RT-PCR (**b**), and flow cytometry (**c**). **a**-**c** All data shown are representative of three independent experiments

To define the signaling pathways involved in TG2-dependent IL-6 expression in breast cancer cells further, TG2 was overexpressed in otherwise TG2- and IL-6-negative luminal-type breast cancer cells (MCF7). The whole sequence of human TG2 was successfully overexpressed (Fig. 1a). Since increased aggressiveness conferred by TG2 expression in breast cancer cells correlates with EMT and stem-cell-like phenotypes, these characteristics were evaluated in TG2 overexpressing cells. Expression of E-cadherin and cell-to-cell junction formation were decreased in TG2-overexpressing MCF7 cells (MCF7_TG2) compared to the control MCF-7 cells (MCF7_Cont) (Fig. 1a and Additional file 1: Figure S1). Snail2, an EMT inducer, and tissue inhibitor of metalloproteinase (TIMP) 1, 2, and 3 were increased in MCF7_TG2 cells compared to the control cells (Fig. 1b). CD44, a breast cancer stem cell surface phenotype marker, was increased in MCF7_TG2 cells compared to control cells (Fig. 1c).

### IL-1β induced IL-6 production from breast cancer cells in a TG2-dependent manner

**Fig. 2:**
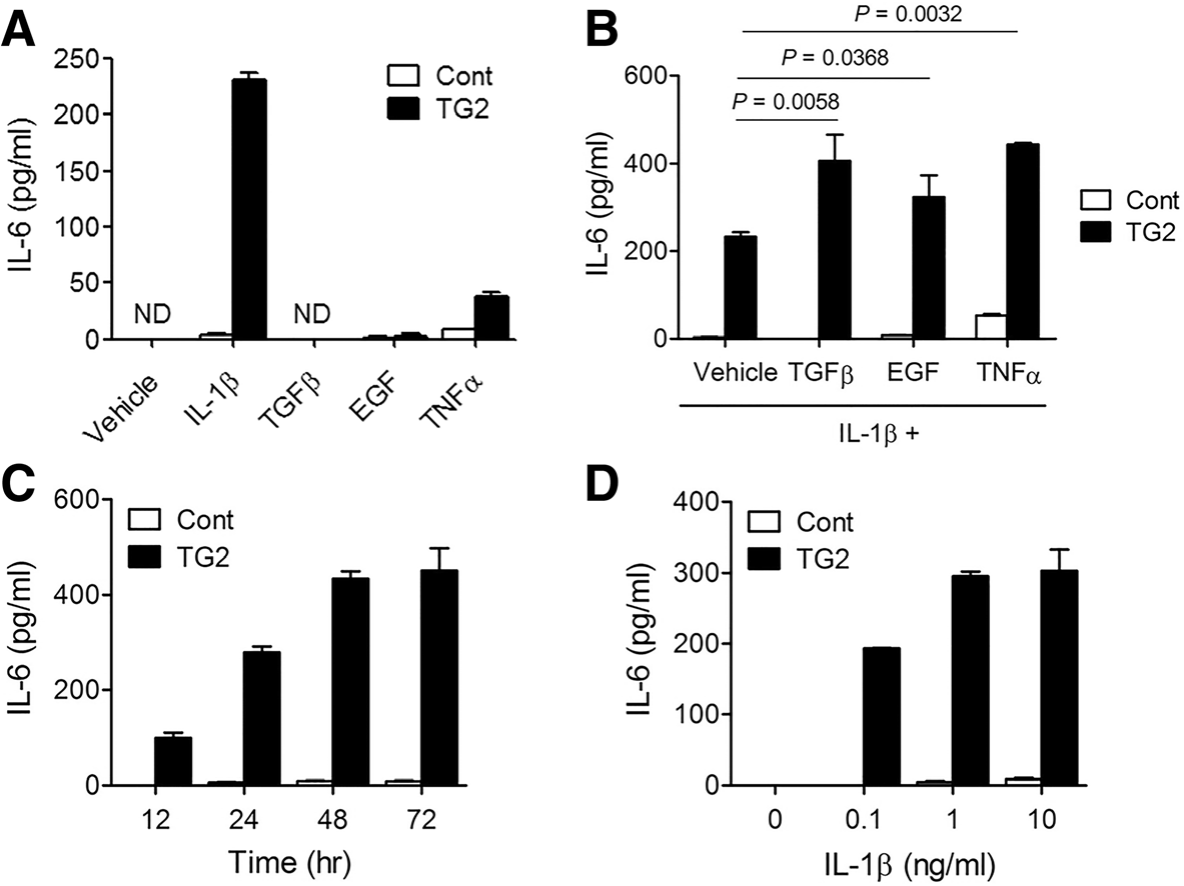
IL-1β induced IL-6 production from breast cancer cells in a TG2-dependent manner. **a** TG2-overexpressing MCF7 cells (TG2) and control vector-transfected MCF-7 cells (Cont) were treated with various cytokines (10 ng/ml) for 48 h and IL-6 levels in culture supernatants were measured by ELISA. **b** Cells were treated with IL-1β (10 ng/ml) in the presence of TGFβ (10 ng/ml), EGF (10 ng/ml), or TNFα (10 ng/ml) for 48 h and secreted IL-6 levels in culture supernatants were measured by ELISA. **c** Cells were treated with IL-1β (10 ng/ml) for the indicated times. **d** Cells were treated with IL-1β at various concentrations for 48 h. **a**-**d** All data shown are representative of three independent experiments. Data are presented as mean ± SD

In our previous report, expression of TG2 and expression of IL-6 were found to correlate with one another, and TG2 was found to promote aggressive phenotypes in breast cancer cells through IL-6. A knockdown (KD) of TG2 in MDA-MB-231 breast cancer cells reduced IL-6 expression, and a knockdown of both TG2 and IL-6 inhibited tumor growth and metastasis [14]. In contrast to our expectations, simple overexpression of TG2 in otherwise TG2- and IL-6-negative luminal-type breast cancer MCF7 cells did not lead to IL-6 expression (Fig. 2a). The behavior and gene expression of cancer cells are affected by the microenvironment surrounding the tumor, and this environment includes cytokines and growth factors released by stromal cells such as leukocytes and fibroblasts. To evaluate the effect of paracrine signals, MCF7 cells were treated with IL-1β, TNF-α, TGF-β, and EGF. The results show that IL-1β induced expression of IL-6 in breast cancer cells, and that TG2 overexpressing cells expressed over twenty times more than control cells after IL-1β treatment. Treating cells with TGF-β or EGF alone did not increase IL-6, but TNF-α treatment slightly increased IL-6 expression (Fig. 2a). Treatment with TGF-β, EGF, and TNF-α after IL-1β further increased IL-6 expression in MCF7_TG2 breast cancer cells (Fig. 2b). Other inflammatory/immune-stimulating reagents, including lipopolysaccharide (LPS), Pam_3_Cys (Pam), peptidoglycan (PGN), CpG, and bleomycin (BLM), did not induce IL-6 expression in either MCF7_Cont or MCF7_TG2 breast cancer cells (Additional file 1: Figure S2).

The mechanism by which IL-1β induces IL-6 expression was evaluated. IL-6 levels were detected in culture supernatants 12 h after treatment, revealing that IL-6 concentrations peaked at from 48 h to 72 h in MCF7_TG2 breast cancer cells (Fig. 2c). The dose-response relationship of IL-1β and IL-6 revealed that as little as 0.1 ng/ml of IL-1β was sufficient to induce the full IL-6 response, and that this response was similar to stimulation with 20 ng/ml of IL-1β (Fig. 2d).

### IL-1β increased stem-cell-like phenotypes, invasion, and estrogen-independent tumor growth of luminal-type breast cancer cells in a TG2-dependent manner

We next evaluated the effect of IL-1β stimulation on MCF7 breast cancer cells. TG2 overexpression in MCF7 cells increased the surface expression of breast cancer stem cell marker CD44, and IL-1β stimulation of MCF7_TG2 breast cancer cells further increased CD44 expression (Additional file 1: Figure S3). CD44 expression increased in a dose-dependent manner after treatment with 0.1 ng/ml to 10 ng/ml of IL-1β stimulation (Additional file 1: Figure S3). Surface expression of CD24 was not changed by IL-1β treatment.

**Fig. 3:**
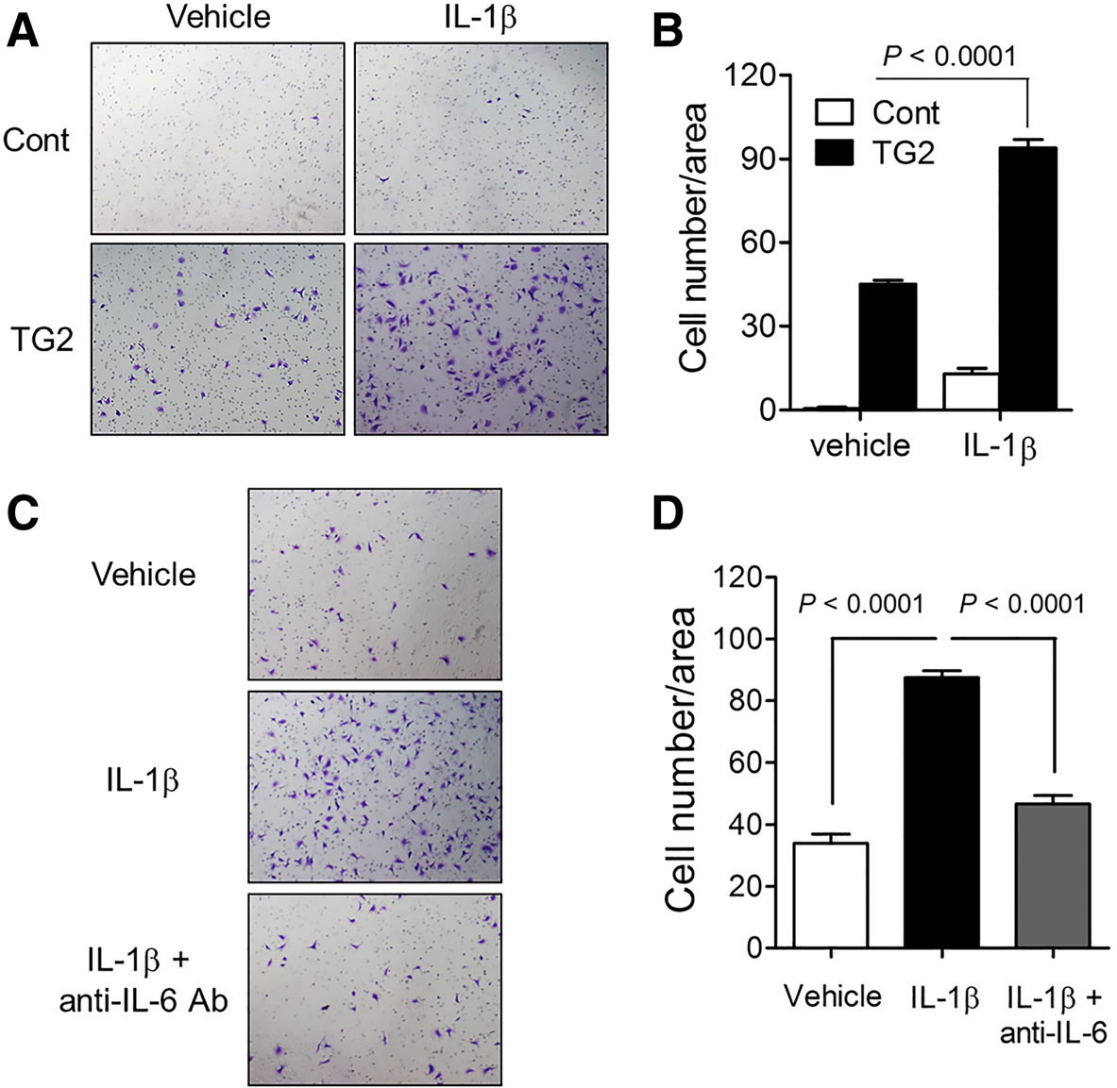
IL-1β increased the invasiveness of luminal-type breast cancer cells in a TG2-dependent manner. **a**-**b** MCF7_Cont and MCF7_TG2 cells were allowed to invade through Matrigel for 48 h in the presence or absence of IL-1β (10 ng/ml). **a** Invaded MCF7_Cont and MCF7_TG2 cells (crystal violet). **b** Invaded cells were counted using ImageJ software. **c**-**d** MCF7_TG2 cells were allowed to invade through Matrigel for 48 h in the presence of IL-1β (10 ng/ml) and anti-IL-6 antibody (10 μg/ml). **c** Invaded MCF7_TG2 cells (crystal violet). **d** Invaded cells were counted using ImageJ software. Data are presented as mean ± SD based on three independent experiments using samples from triplicate cell cultures

**Fig. 4:**
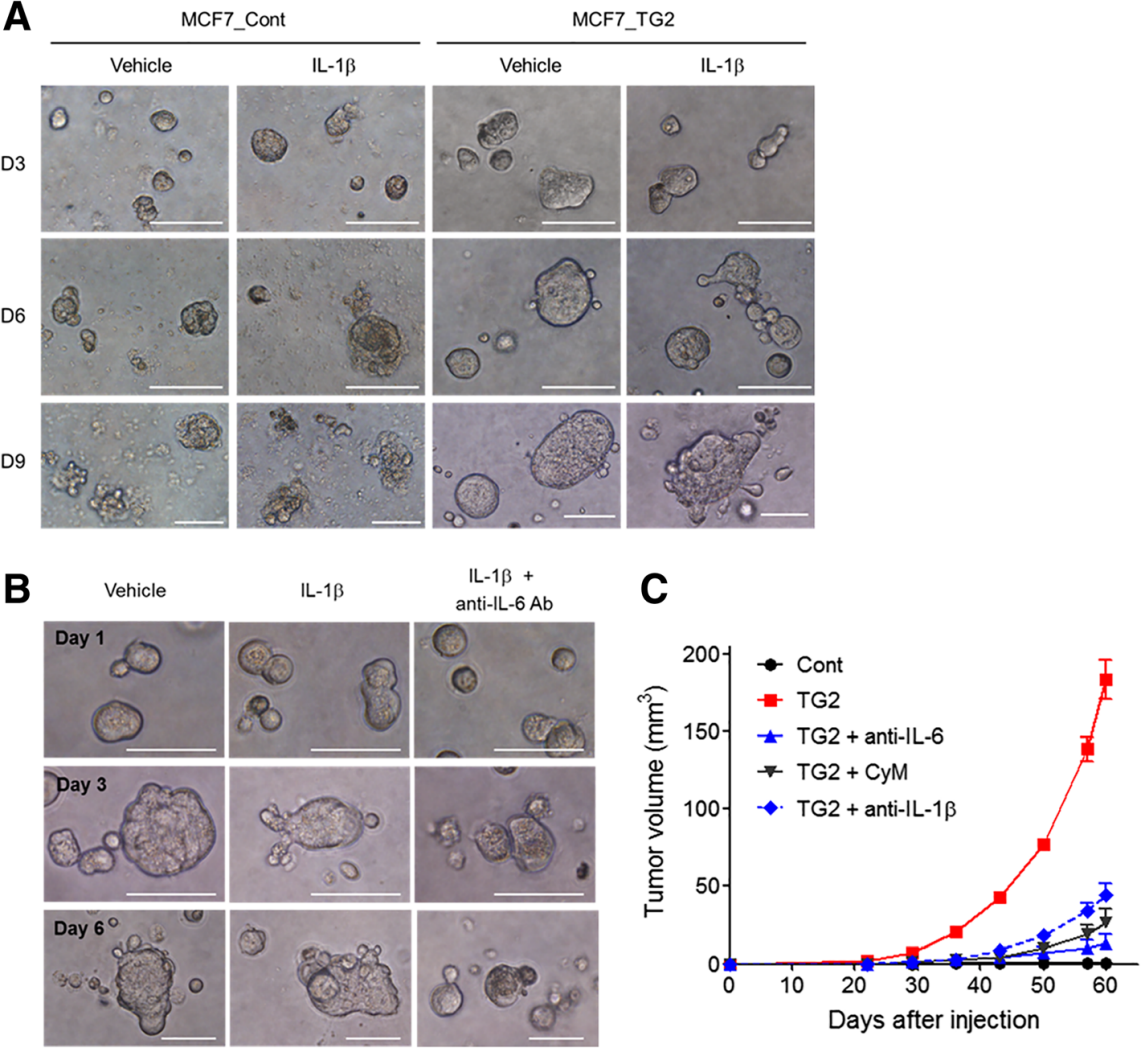
Three-dimensional culture resulted in dramatically enhanced survival of luminal-type breast cancer cells in a TG2-dependent manner. **a** MCF7_Cont and MCF7_TG2 cells were grown in 3D culture conditions in the presence or absence of IL-1β (10 ng/ml). **b** MCF7_TG2 cells were grown in 3D culture conditions in the presence or absence of IL-1β (10 ng/ml) and anti-IL-6 monoclonal antibody (10 μg/ml). **c** MCF7_Cont and MCF7_TG2 cells (1 × 10^6^ cells/each mouse) were injected into the fat pads of NSG mice. Primary tumor growth was measured. Blocking anti-IL-6 antibody (100 μg/mouse) or blocking anti-IL-1β antibody (100 μg/mouse) was injected intraperitoneally every third day, starting 1 day after tumor inoculation. The TG2 inhibitor cysteamine (CyM, 40 mg/kg/day) was injected intraperitoneally starting 1 day after tumor inoculation. Data are given as mean ± SEM of six mice for each group

To evaluate the biological behavior of TG2 overexpression and IL-1β stimulation in breast cancer cells, a two-dimensional (2D) matrigel invasion assay was performed. MCF7_TG2 cells showed increased invasiveness compared to MCF7_Cont cells, and IL-1β treatment further increased the invasiveness of MCF7_TG2 breast cancer cells (Fig. 3a and b). Increased invasion of MCF7_TG2 breast cancer cells by IL-1β was attenuated by blocking IL-6 through anti-IL-6 antibody treatment (Fig. 3c and d). A 3D matrigel on top assay also revealed the synergistic effects of TG2 overexpression and IL-1β treatment on the invasion of MCF7 breast cancer cells. MCF7_TG2 breast cancer cells grew more rapidly and formed a large spheroid in the 3D matrigel compared to MCF7_Cont cells, and IL-1β treatment further increased growth and conferred invasiveness and budding-like phenomena in MCF7_TG2 cells (Fig. 4a). Again, these aggressive phenotypes were ameliorated by anti-IL-6 antibody treatment (Fig. 4b). Moreover, an in vivo tumorigenesis assay in NSG mice revealed that, unlike estrogen-dependent MCF7_Cont cells, MCF7_TG2 breast cancer cells obtained tumorigenic capability in vivo without the addition of exogenous estrogen, which were reduced in the presence of blocking anti-IL-6 or anti-IL-1β antibodies or the TG2 inhibitor cysteamine (CyM) (Fig. 4c).

### IL-1β induced IL-6 production from TG2 overexpressed breast cancer cells in an NF-kB-, JNK-, and PI3K-dependent manner

**Fig. 5:**
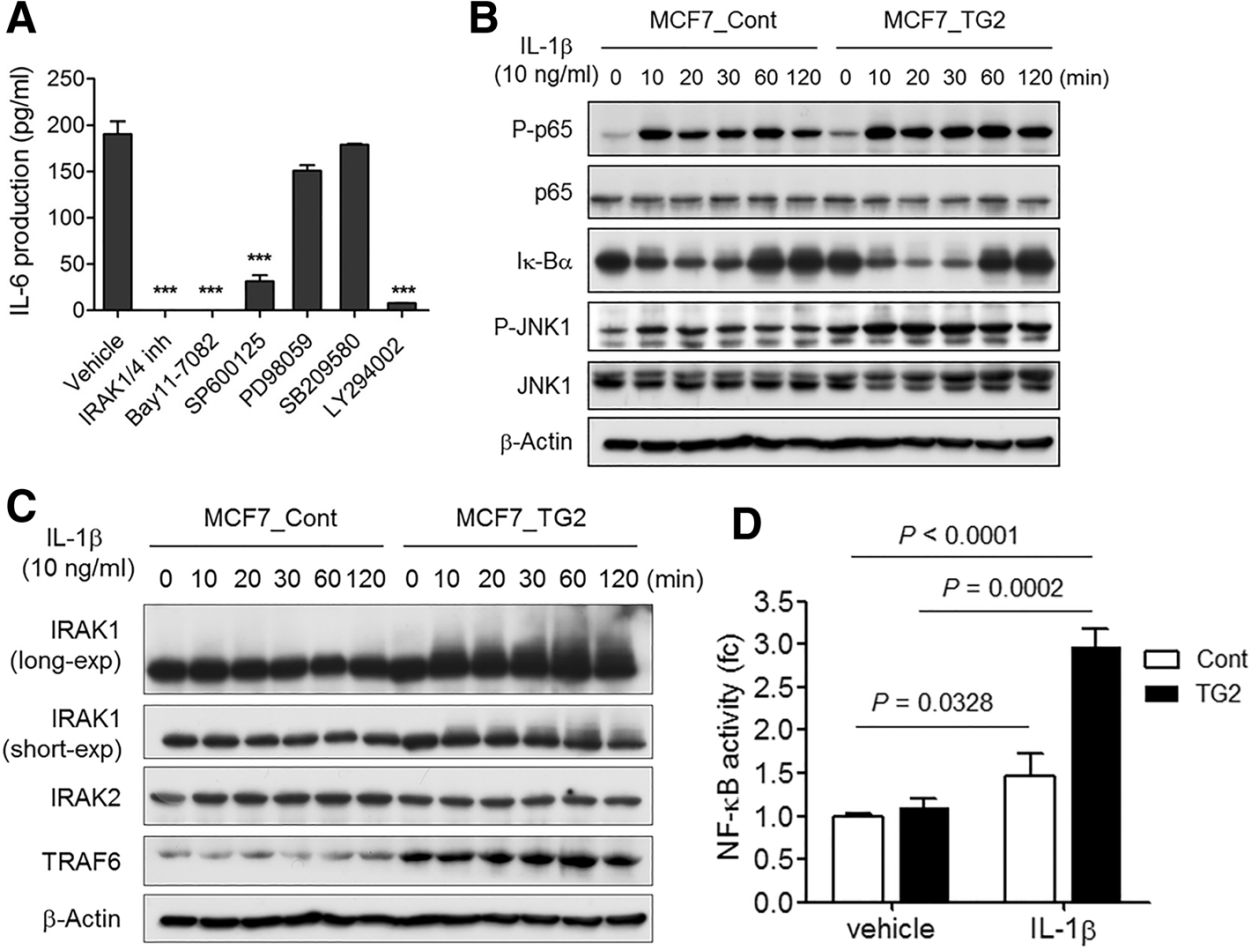
IL-1β induced IL-6 production in MCF7_TG2 cells through the IRAK1, NF-kB, JNK, and PI3K signaling pathway. **a** MCF7_TG2 cells were treated with IL-1β (10 ng/ml) in the presence of IRAK1/4 inhibitor (20 μM), a NF-kB inhibitor (Bay11-7082, 10 μM), a JNK inhibitor (SP600125, 10 μM), an ERK inhibitor (PD98059, 10 μM), a p38 MAPK inhibitor (SB209580, 10 μM), or a PI3K inhibitor (LY294002, 10 μM) for 48 h. IL-6 levels in culture supernatants were measured by ELISA. **b** MCF7_Cont and MCF7_TG2 cells were treated with IL-1β (10 ng/ml) for the indicated times. Phospho-p65, p65, Ik-Bα, phospho-JNK, and JNK were detected by Western blot. **c** MCF7_Cont and MCF7_TG2 cells were treated with IL-1β (10 ng/ml) for the indicated times. IRAK1, IRAK2, and TRAF6 were detected by Western blot. **d** MCF7_Cont and MCF7_TG2 cells were co-transfected with p3kB-Luc and pRL-TK reporter constructs for 24 h then treated with IL-1β (10 ng/nl) for 18 h. All data shown are representative of three independent experiments

To evaluate the underlying molecular mechanisms by which TG2- and IL-1β-dependent IL-6 production results in increased stem-cell characteristics, invasiveness, and hormone-independent in vivo tumorigenesis, we evaluated the key signaling pathways downstream of TG2 and IL-1β by utilizing several signaling pathway inhibitors. IL-6 expression from IL-1β stimulated TG2-overexpressing MCF7_TG2 breast cancer cells was inhibited by an IRAK1/4 inhibitor, BAY11-7082 (a NF-kB inhibitor), SP600125 (a JNK inhibitor), and LY294002 (a PI3K inhibitor), but not by PD98059 (an ERK inhibitor) or SB209580 (a p38 mitogen-activated protein kinase inhibitor) (Fig. 5a). Western blot analysis revealed that activated JNK1 kinase, phospho-JNK1 (P-JNK1), levels were increased by IL-1β stimulation in MCF7_TG2 cells compared to MCF7_Cont cells (Fig. 5b). Basal levels of NF-kB signaling molecules, including p65, phospho-p65, and IkBα, were not changed by TG2 overexpression in MCF7 breast cancer cells (Fig. 5b). IL-1β treatment decreased IkB levels and increased phospho-p65 levels within 10 min of IL-1β stimulation. IkBα levels normalized starting at 60 min post treatment, and phospho-p65 levels began to decrease after 120 min in both MCF7_Cont and MCF7_TG2 breast cancer cells (Fig. 5b). The IL-1β treatment-induced IkBα decrease and the phospho-p65 increase were slightly greater in MCF7_TG2 cells compared to MCF7_Cont cells (Fig. 5b). NF-kB activity was measured using a luciferase reporter assay and found to be increased by IL-1β treatment in MCF7_TG2 and MCF7_Cont cells; however, the degree of increase was greater in MCF7_TG2 cells (Fig. 5d). These results suggest that increased activation of NF-kB signaling by IL-1β in the presence of TG2 is necessary to induce IL-6 expression in MCF7 breast cancer cells.

**Fig. 6:**
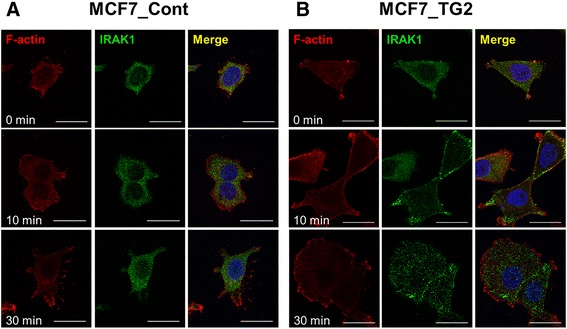
Confocal microscopic analysis of IRAK1 following IL-1β stimulation. **a**-**b** MCF7_Cont (**a**) and MCF7_TG2 (**b**) cells were treated with IL-1β (10 ng/ml) for the indicated times. Cells were stained for IRAK1 (green), F-actin (red), and DAPI (blue) to compare their cellular locations. All data shown are representative of three independent experiments. Scale bar = 30 μm (original magnification, ×1000)

We next evaluated the signaling molecules downstream of IL-1 receptors. Among others, TRAF6 expression was increased by TG2 overexpression, and MCF7_TG2 cells had higher levels of TRAF6 protein compared to MCF7_Cont cells (Fig. 5c). However, TRAF6 levels were not increased by IL-1β stimulation. Although the expression of IRAK1 and IRAK2 were not changed by TG2 overexpression, modified IRAK1, characterized by increased molecular weight, was more evident in MCF7_TG2 cells compared to MCF7_Cont cells following IL-1β stimulation (Fig. 5c). Modified IRAK1 levels peaked after 60 min of IL-1β stimulation and IRAK1 levels decreased thereafter and remained low until 24 h post stimulation (Fig. 5c and Additional file 1: Figure S4). Since TG2 is also expressed in the nucleus, we evaluated whether TG2 directly interacted with IL-1 receptor signaling molecules. The extracts from MCF7_TG2 cells were pulled down with anti-TG2 antibody and immunoblotted with anti-TG2, anti-IRAK1, anti-TRAF6, and anti-MyD88 antibodies, but TG2 molecules were not directly bound to any of the IL-1-receptor signaling molecules tested (Additional file 1: Figure S5). Confocal microscopic analysis revealed that TG2-overexpressing cells showed increased accumulation of IRAK1 in the plasma membrane from 10 min following IL-1β stimulation, and increased cytoplasmic localization of IRAK1 from 30 min of stimulation compared to MCF7_Cont cells (Fig. 6).

## Discussion

The tumor microenvironment, in particular the inflammatory environment, has been shown to affect cancer cell behavior, including cancer formation, invasion and metastasis [19]. Studies on several types of cancer associated with infection, including *Helicobacter pylori*, hepatitis B and C, and chronic inflammation such as Crohn’s disease, have proven the inflammation-induced oncogenesis hypothesis [20]. In addition, established cancer cells recruit various inflammatory cells and signal them not to attack the cancer [21]; cancer cells also utilize prototypical inflammatory signaling pathways [22]. The interdependent activation of two inflammatory signaling pathways, NF-kB and STAT3, in this study resulted in increased breast cancer cell aggressiveness and hormone-independent tumor growth.

IL-1β, a prototypical inflammatory cytokine, is induced and activated following infection and tissue damage. High IL-1 levels in the tumor microenvironment are associated with a more aggressive tumor phenotype and generally poor prognoses [9]. In obesity, IL-1 and leptin production are increased while adiponectin production is decreased, which may be the reason that obesity increases breast cancer risk [23]. Although pro-inflammatory cytokines such as IL-1β and TNFα induce IL-6 production from innate immune cells during acute inflammation, this is not the case in human breast cancer cells. Treatment of luminal-type breast cancer cells with IL-1β did not induce IL-6 production in this study. In our previous report, IL-6 was expressed in more aggressive breast cancer cells, including basal-like cells, and TG2 was the upstream molecule that induced IL-6 production in these cells [17]. Thus, the effect of IL-1β on TG2-overexpressing luminal-type breast cancer cells, which do not normally express TG2 or IL-6, was evaluated. IL-1β treatment induced IL-6 production in TG2-overexpressing breast cancer cells and resulted in an aggressive phenotype that showed increased invasion, EMT phenotypes, and cancer stem-cell-like properties. Moreover, tumor formation from TG2-overexpressing breast cancer cells was found to be hormone-independent in immunocompromised mice. Although IL-1β was not applied during in vivo tumor formation, many innate inflammatory cells, including macrophages and myeloid-derived suppressor cells (MDSCs) were recruited to the tumor sites in the host mice (data not shown). Therefore, recruited inflammatory cells in vivo would provide IL-1β or equivalent signals in the tumor microenvironment to induce hormone-independent tumorigenesis. When the IL-1 receptor signaling pathway was examined, TG2 expression was found to increase TRAF6 expression, IRAK1 modification, and IRAK1 plasma membrane accumulation and IRAK1 nuclear translocation, and thus improved IL-1β signaling efficiency leading to IL-6 production [24, 25]. In terms of the relevance of IL-1β to breast cancer cell aggressiveness, breast cancer metastasis suppressor 1 has been shown to up-regulate miR-146, which targets key IL-1 receptor signaling molecules, including IRAK1 and TRAF6, and suppresses the metastasis of breast cancer cells [26].

IL-6 is a critical link between inflammation and cell transformation in mammary tissue [27], and IL-6 signaling in cancer cells results in EMT phenotypes that facilitate cancer cell invasion into the surrounding tissue and blood vessels, and cause distant metastasis [10, 17]. IL-6 is also a critical survival signal in breast CSCs, and switches the dynamic equilibrium toward breast CSCs over non-stem cell like cancer cells, which leads to in vivo tumorigenesis, drug resistance, and recurrence [12, 13]. The positive feedback loop of two key inflammatory signaling pathways, NF-kB and IL-6/STAT3, has been suggested as a link between inflammation and cancer in previous studies [27, 28]. In this respect, since, through IL-1β signaling, TG2 is an activator of NF-kB [29, 30] and a critical mediator of IL-6 production in luminal-type breast cancer, and since TG2 is highly expressed in drug-resistant cancer cells [31], TG2 may function as a component of the positive feedback loop between NF-kB and IL-6/STAT3, which mediates cancer aggressiveness and hormone-independent tumor growth. Therefore, IL-1β in the tumor microenvironment and tumor cell expression of TG2 may be potential targets for combating resistance in luminal-type breast cancer cells. Since IL-6/STAT3 is also an important mediator of the breast cancer cell-MDSC positive signaling loop [32], targeting key molecules in the IL-6/STAT3 pathways may be a promising therapy for recurrent and drug resistant breast cancers [33].

## Conclusions

Our findings indicate that luminal-type breast cancer cells acquire the ability to produce IL-6 through cancer cell TG2 overexpression and IL-1β from the tumor microenvironment. Unlike inflammatory cells, IL-1β stimulation did not lead to IL-6 production, and the overexpression of TG2 alone did not lead to IL-6 production in luminal-type breast cancer cells. In breast cancer cells, TG2 overexpression conferred EMT and stem cell-like phenotypes, while IL-1β treatment increased stem cell-like phenotypes, cell invasion, and estrogen-independent tumor growth in a TG2-dependent manner; however, these effects were attenuated by treatment with either anti-IL-6 or anti-IL-1β antibodies. IL-1β induced IL-6 production from TG2-overexpressing MCF7 breast cancer cells in an NF-kB-, PI3K-, and JNK-dependent manner. Thus, within the inflammatory tumor microenvironment, IL-1β, together with other cytokines and growth factors, increases breast cancer cell aggressiveness in a TG2-dependent manner. Therefore, IL-1β in the tumor microenvironment and tumor cell TG2 and IL-6/STAT3 signaling pathway may be potential targets for combating recurrent and therapy-resistant luminal-type breast cancer.

## Additional file

Additional file 1:
**Figure S1.** Representative photos of TG2-overexpressing MCF7 cells (MCF7_TG2) and control vector-transfected MCF-7 cells (MCF7_Cont). **Figure S2.** Effect of immunostimulants on TG2-overexpressing MCF7 cell (TG2) and control vector-transfected MCF-7 cell (Cont) IL-6 expression as measured by ELISA. Cells were treated with toll-like receptor agonists; lipopolysaccharide (LPS, 1 μg/ml), Pam_3_Cys (Pam, 5 μg/ml), peptidoglycan (PGN, 50 μg/ml), and CPG-ODN (CPG, 1 mM), and bleomycin (BLM, 1 μg/ml) for 48 h. **Figure S3.** IL-1β increased stem-cell-like phenotypes in a TG2-dependent manner. MCF7_Cont and MCF7_TG2 cells were treated with IL-1β for 6 days, and cell surface expression of CD24 and CD44 was analyzed by flow cytometry analysis. **Figure S4.** MCF7_Cont and MCF7_TG2 cells were treated with IL-1β (10 ng/ml) for the indicated times. IRAK1 and IRAK2 were detected by Western blot. **Figure S5.** MCF7_TG2 cells were treated with IL-1β (10 ng/ml) for 30 min. Cell lysates were immunoprecipitated with an anti-TG2 antibody, and TG2, IRAK1, TRAF6, and MyD88 were detected by Western blot. (DOC 1319 kb)

## Abbreviations

2D: Two-dimensional
3D: Three-dimensional
ANOVA: Two-way analysis of variance
BLM: Bleomycin
CSC: Cancer stem cell
DMEM: Dulbecco’s Modified Eagle Medium
EMT: Epithelial-mesenchymal transition
ER: Estrogen receptor
FBS: Fetal bovine serum
IL-6: Interleukin-6
KD: Knockdown
LPS: Lipopolysaccharide
MDSC: Myeloid-derived suppressor cells
M-MLV: Moloney murine leukemia virus
NOG: NOD/scid/IL-2Rγ^−/−^
Pam: Pam_3_Cys
PGN: Peptidoglycan
P-JNK1: Phospho-JNK1
TG2: Transglutaminase 2
TIMP: Tissue inhibitor of metalloproteinase
