# Supplementary material for: IL-1β induces IL-6 production and increases invasiveness and estrogen-independent growth in a TG2-dependent manner in human breast cancer cells
Source: BMC Cancer. 2016 Sep 8;16(1):724. doi: 10.1186/s12885-016-2746-7 (PMC5017052; doi:10.1186/s12885-016-2746-7)
Supplement: Additional file 1: — Figure S1. Representative photos of TG2-overexpressing MCF7 cells (MCF7_TG2) and control vector-transfected MCF-7 cells (MCF7_Cont). Figure S2. Effect of immunostimulants on TG2-overexpressing MCF7 cell (TG2) and control vector-transfected MCF-7 cell (Cont) IL-6 expression as measured by ELISA. Cells were treated with toll-like receptor agonists; lipopolysaccharide (LPS, 1 μg/ml), Pam3Cys (Pam, 5 μg/ml), peptidoglycan (PGN, 50 μg/ml), and CPG-ODN (CPG, 1 mM), and bleomycin (BLM, 1 μg/ml) for 48 h. Figure S3. IL-1β increased stem-cell-like phenotypes in a TG2-dependent manner. MCF7_Cont and MCF7_TG2 cells were treated with IL-1β for 6 days, and cell surface expression of CD24 and CD44 was analyzed by flow cytometry analysis. Figure S4. MCF7_Cont and MCF7_TG2 cells were treated with IL-1β (10 ng/ml) for the indicated times. IRAK1 and IRAK2 were detected by Western blot. Figure S5. MCF7_TG2 cells were treated with IL-1β (10 ng/ml) for 30 min. Cell lysates were immunoprecipitated with an anti-TG2 antibody, and TG2, IRAK1, TRAF6, and MyD88 were detected by Western blot. (DOC 1319 kb) [file 12885_2016_2746_MOESM1_ESM.doc]

**Additional file 1**

**
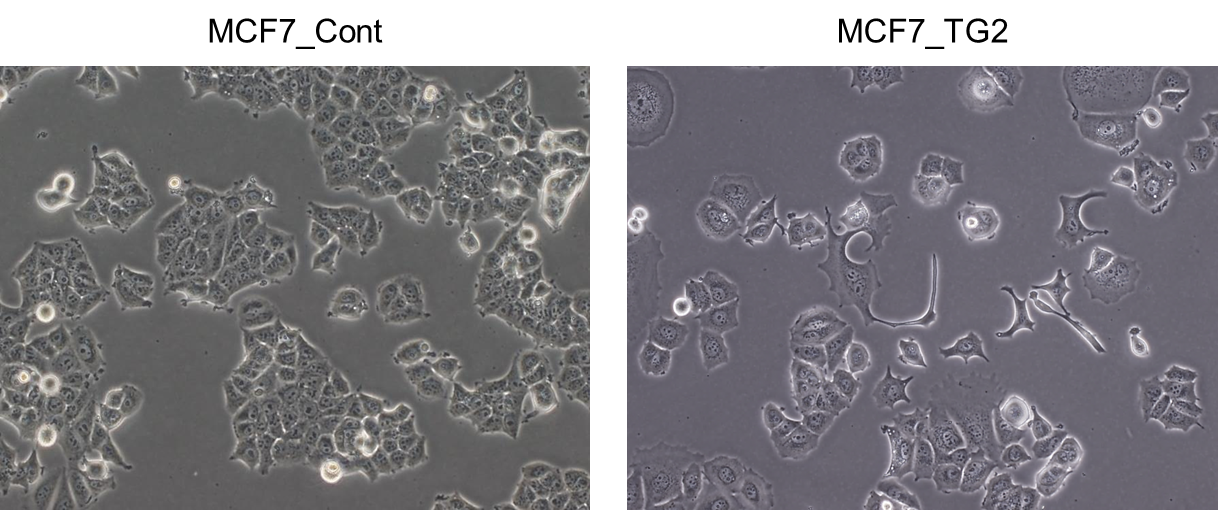
**

**Figure S1**. Representative photos of TG2-overexpressing MCF7 cells (MCF7_TG2) and control vector-transfected MCF-7 cells (MCF7_Cont).

**
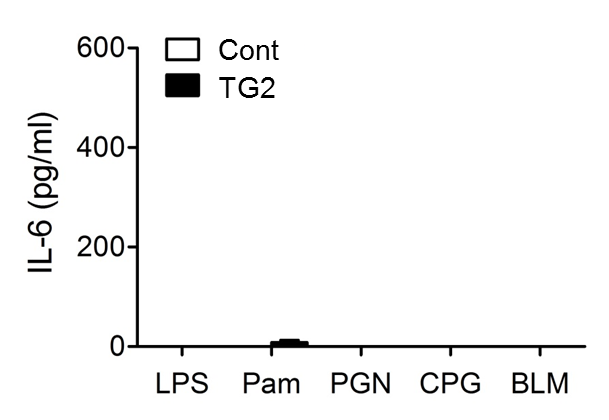
**

**Figure S2**. Effect of immunostimulants on TG2-overexpressing MCF7 cell (TG2) and control vector-transfected MCF-7 cell (Cont) IL-6 expression as measured by ELISA. Cells were treated with toll-like receptor agonists; lipopolysaccharide (LPS, 1 μg/ml), Pam_3_Cys (Pam, 5 μg/ml), peptidoglycan (PGN, 50 μg/ml), and CPG-ODN (CPG, 1 mM), and bleomycin (BLM, 1 μg/ml) for 48 h.


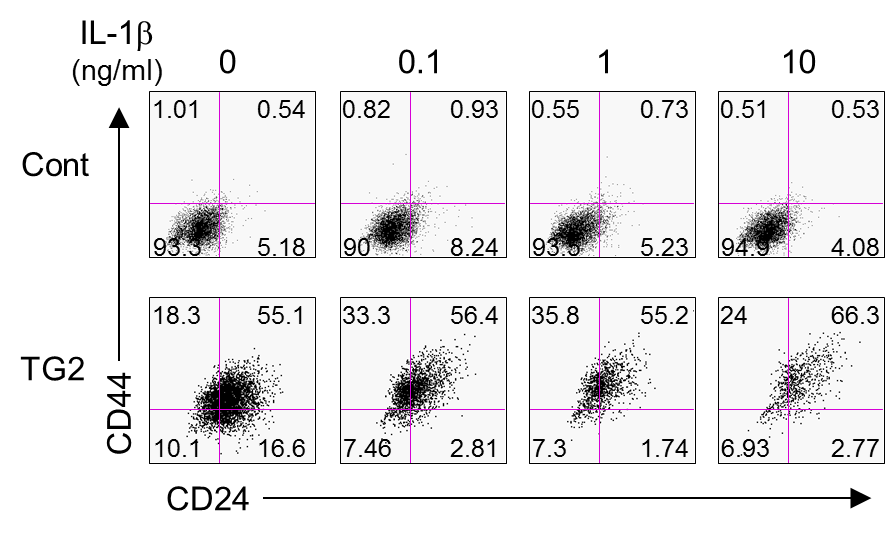


**Figure S3**. **IL-1**β **increased stem-cell-like phenotypes in a TG2-dependent manner.** MCF7_Cont and MCF7_TG2 cells were treated with IL-1β for 6 days, and cell surface expression of CD24 and CD44 was analyzed by flow cytometry analysis.


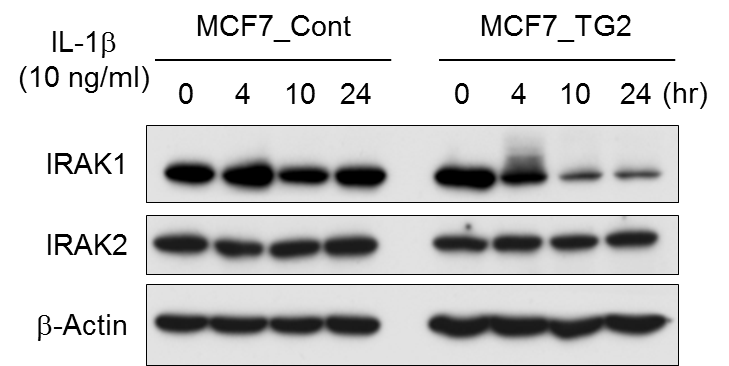


**Figure S4**. MCF7_Cont and MCF7_TG2 cells were treated with IL-1β (10 ng/ml) for the indicated times. IRAK1 and IRAK2 were detected by Western blot.


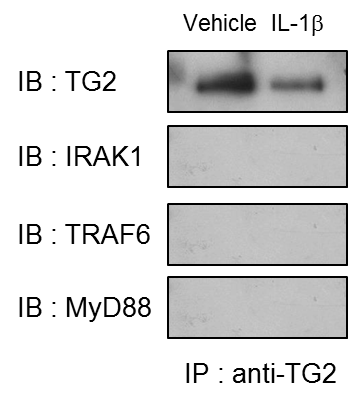


**Figure S5**. MCF7_TG2 cells were treated with IL-1β (10 ng/ml) for 30 min. Cell lysates were immunoprecipitated with an anti-TG2 antibody, and TG2, IRAK1, TRAF6, and MyD88 were detected by Western blot.
